# Exploring the ultrafast and isomer-dependent photodissociation of iodothiophenes *via* site-selective ionization†

**DOI:** 10.1039/d3cp06079a

**Published:** 2024-04-05

**Authors:** Weronika O. Razmus, Felix Allum, James Harries, Yoshiaki Kumagai, Kiyonobu Nagaya, Surjendu Bhattacharyya, Mathew Britton, Mark Brouard, Philip H. Bucksbaum, Kieran Cheung, Stuart W. Crane, Mizuho Fushitani, Ian Gabalski, Tatsuo Gejo, Aaron Ghrist, David Heathcote, Yasumasa Hikosaka, Akiyoshi Hishikawa, Paul Hockett, Ellen Jones, Edwin Kukk, Hiroshi Iwayama, Huynh V. S. Lam, Joseph W. McManus, Dennis Milesevic, Jochen Mikosch, Shinichirou Minemoto, Akinobu Niozu, Andrew J. Orr-Ewing, Shigeki Owada, Daniel Rolles, Artem Rudenko, Dave Townsend, Kiyoshi Ueda, James Unwin, Claire Vallance, Anbu Venkatachalam, Shin-ichi Wada, Tiffany Walmsley, Emily M. Warne, Joanne L. Woodhouse, Michael Burt, Michael N. R. Ashfold, Russell S. Minns, Ruaridh Forbes

**Affiliations:** a School of Chemistry, University of Southampton, Highfield Southampton SO17 1BJ UK r.s.minns@soton.ac.uk; b Chemistry Research Laboratory, Department of Chemistry, University of Oxford Oxford OX1 3TA UK; c PULSE Institute, SLAC National Accelerator Laboratory 2575 Sand Hill Road Menlo Park CA 94025 USA; d Linac Coherent Light Source, SLAC National Accelerator Laboratory 2575 Sand Hill Road Menlo Park CA 94025 USA ruforbes@stanford.edu; e QST, SPring-8, Kouto 1-1-1 Sayo Hyogo Japan; f Department of Applied Physics, Tokyo University of Agriculture and Technology Tokyo Japan; g Department of Physics, Kyoto University Kyoto 606-8502 Japan; h J.R. Macdonald Laboratory, Department of Physics, Kansas State University Manhattan Kansas 66506 USA; i Institute of Photonics and Quantum Sciences, Heriot-Watt University Edinburgh EH14 4AS UK; j Department of Chemistry, Graduate School of Science, Nagoya University Furo-cho, Chikusa Nagoya Aichi 464-8602 Japan; k Graduate School of Material Science, University of Hyogo Kuoto 3-2-1, Kamigori-cho, Ako-gun Hyogo 678-1297 Japan; l Department of Applied Physics, Stanford University Stanford California 94305 USA; m Institute of Liberal Arts and Sciences, University of Toyama Toyama 930-0194 Japan; n Research Center for Materials Science, Nagoya University Furo-cho, Chikusa Nagoya Aichi 464-8602 Japan; o National Research Council of Canada 100 Sussex Dr Ottawa ON K1A 0R6 Canada; p Department of Physics and Astronomy, University of Turku FI-20014 Turku Finland; q Institute for Molecular Science Okazaki 444-8585 Japan; r Department of Physics, University of Kassel Heinrich-Plett-Strasse 40 34132 Kassel Germany; s Department of Physics, Graduate School of Science, The University of Tokyo 7-3-1 Hongo, Bunkyo-ku Tokyo 113-0033 Japan; t Graduate School of Advanced Science and Engineering, Hiroshima University Higashi-Hiroshima 739-8526 Japan; u School of Chemistry, University of Bristol Cantock's Close Bristol BS8 1TS UK; v RIKEN SPring-8 Center Sayo Hyogo 679-5148 Japan; w Japan Synchrotron Radiation Research Institute Hyogo Japan; x Department of Chemistry, Tohoku University Sendai 980-8578 Japan; y Department of Condensed Matter Physics and Photon Science, School of Physical Science and Technology, ShanghaiTech University Shanghai 201210 China

## Abstract

C–I bond extension and fission following ultraviolet (UV, 262 nm) photoexcitation of 2- and 3-iodothiophene is studied using ultrafast time-resolved extreme ultraviolet (XUV) ionization in conjunction with velocity map ion imaging. The photoexcited molecules and eventual I atom products are probed by site-selective ionization at the I 4d edge using intense XUV pulses, which induce multiple charges initially localized to the iodine atom. At C–I separations below the critical distance for charge transfer (CT), charge can redistribute around the molecule leading to Coulomb explosion and charged fragments with high kinetic energy. At greater C–I separations, beyond the critical distance, CT is no longer possible and the measured kinetic energies of the charged iodine atoms report on the neutral dissociation process. The time and momentum resolved measurements allow determination of the timescales and the respective product momentum and kinetic energy distributions for both isomers, which are interpreted in terms of rival ‘direct’ and ‘indirect’ dissociation pathways. The measurements are compared with a classical over the barrier model, which reveals that the onset of the indirect dissociation process is delayed by ∼1 ps relative to the direct process. The kinetics of the two processes show no discernible difference between the two parent isomers, but the branching between the direct and indirect dissociation channels and the respective product momentum distributions show isomer dependencies. The greater relative yield of indirect dissociation products from 262 nm photolysis of 3-iodothiophene (*cf.* 2-iodothiophene) is attributed to the different partial cross-sections for (ring-centred) π∗ ← π and (C–I bond localized) σ∗ ← (n/π) excitation in the respective parent isomers.

## Abstract 2

The dissociation dynamics of UV pumped iodothiophene molecules are investigated using velocity map ion imaging, in combination with site-selective extreme ultraviolet ionization of the iodine atom.

## Graphical abstract

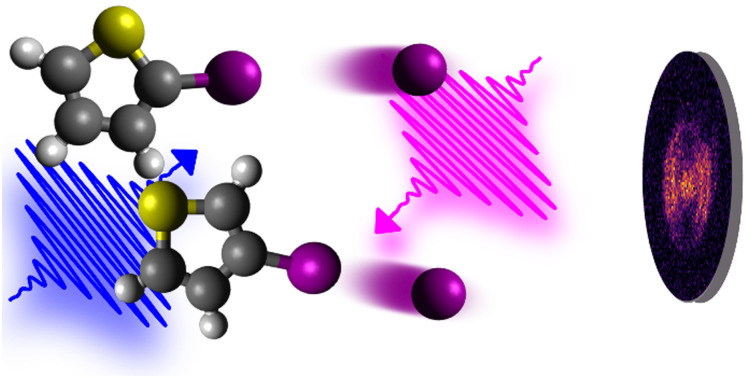

## I. Introduction

Fundamental photochemical processes such as photolysis continue to be the subject of intense study. This seemingly simple chemical change is often the consequence of complex electronic and geometric structure rearrangements that are a challenge to measure experimentally, and to model theoretically. The photodissociation of alkyl halides following ultraviolet (UV) excitation within the so-called A band continuum constitute benchmark systems for study,^1–3^ with ultrafast breaking of the carbon–halogen (C–X) bond seen in conjunction with non-adiabatic transitions between excited states arising from excitation of an electron from the non-bonding (n) highest occupied molecular orbital in the ground state to an anti-bonding σ* orbital localised on the C–X bond.^4,5^ Many such investigations of alkyl halide photolysis have exploited Resonance Enhanced Multiphoton Ionization (REMPI) in conjunction with Velocity Map Imaging (VMI) detection.^6,7^ REMPI detection enables quantum state-selective measurements of the chosen photofragment and determination of its momentum relative to the laser polarization axis, *i.e.* its recoil velocity distribution and, by energy conservation, the internal (rotation and vibration) energy partitioning within both fragments. In the case of methyl iodide, for example, both ground (I) and spin–orbit excited (I*) iodine fragments are formed following excitation within its A band (spanning the wavelength range ∼230–290 nm^8^), with preferential parallel recoil anisotropy. Most of the excess energy (*i.e.* the photon energy in excess of the C–I bond dissociation energy) is released as fragment kinetic energy.^6^ For more complex haloalkanes, increasing portions of this excess energy are also partitioned into internal modes of the fragments.^9^ Photodissociation experiments performed with femtosecond photolysis and REMPI probe lasers can also reveal the time taken for the C–X bond to break, often termed ‘clocking’ the dissociation timescale.^10^

**Fig. 1:**
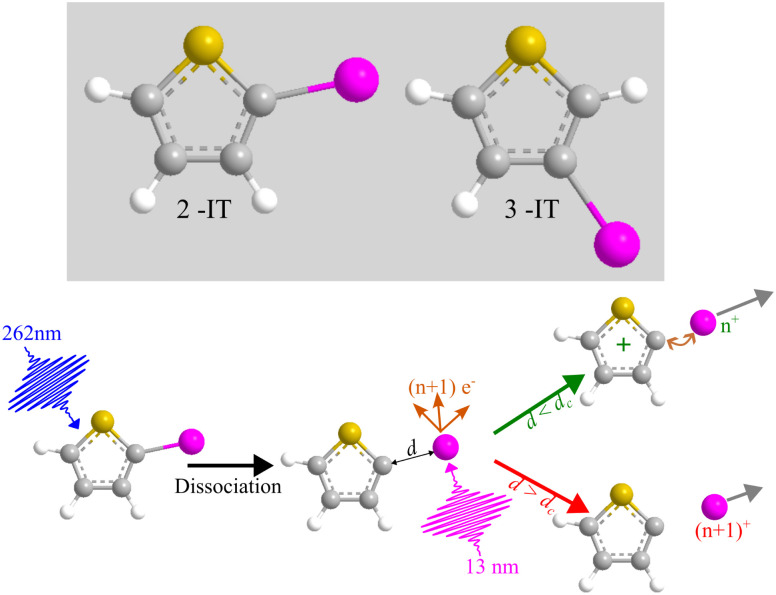
Top: A ball and stick representation of 2-iodothiophene (2IT) and 3-iodothiophene (3IT). In both structures carbon atoms are gray, hydrogen is white, iodine is purple and sulfur is yellow. Bottom: A schematic representation of the measurement process. 262 nm light absorption leads to fission of the C–I bond. Subsequent site-selective ionization with a 13 nm pulse leads to an (*n* + 1)^+^ charge on the iodine atom. If the separation between the co-fragments, *d*, is less than the critical distance, *d*_c_, charge can transfer to the neutral thiophenyl co-fragment leading to Coulomb explosion. Once the distance is greater than *d*_c_, CT can no longer occur, and the kinetic energy of the iodine ion measured is determined by the neutral dissociation process.

Alternative routes to exploring molecular photodissociation processes (both the timescales and the product energy disposal) based on coupling ultrafast UV excitation with the ultrafast ionization of a specific atomic site within the target molecule using light from extreme ultraviolet (XUV) free-electron laser (FEL) sources are now emerging.^11–15^ These experiments exploit the strong wavelength dependencies of different atomic ionization cross-sections in the XUV and X-ray regions of the electromagnetic spectrum, which ensure selective ionization of particular atomic sites within molecules.^16,17^ Fast Auger–Meitner decay results in multiple charges localized on the target atomic site, which may subsequently redistribute around the rest of the molecule. Such charge redistribution has a strong distance dependence that can be exploited to extract details of neutral photodissociation processes. Several ultrafast pump–probe studies based on UV induced C–I bond fission in an alkyl iodide and subsequent XUV ionization of the iodine atom have explored the distance dependence of the CT process and the point at which CT can no longer occur.^11,13,18,19^ A schematic representation of the processes occurring in these measurements is presented in Fig. 1 and is often described using a classical over-the-barrier (OTB) model,^18,20,21^ which defines a critical C–I distance (*d*_c_) beyond which CT is forbidden. The value of *d*_c_ can be calculated using eqn (1).1
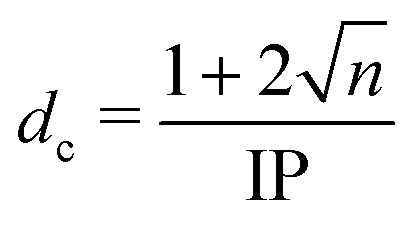
where *n* is the iodine charge state and IP is the ionization energy of the neutral co-fragment. Note that eqn (1) is in atomic units. If the iodine atom is ionized at separations less than *d*_c_, CT can occur and the resulting charged moieties will repel one another: the molecule will undergo Coulomb explosion leading to fragments with high momenta. For ionization at distances greater than *d*_c_, however, CT from the charged iodine atom cannot occur and the molecule will no longer undergo Coulomb explosion (provided that the partner fragment only has a very small ionization cross-section at the chosen XUV probe wavelength). The OTB model thus provides a useful physical picture for describing how *d*_c_ changes with, for example, the charge state *n*. The key observable for the cessation of CT is the appearance of charged atomic iodine fragments with constant and relatively low momenta. As the I^*n*+^ ion can no longer redistribute charge to the rest of the molecule, it does not gain any kinetic energy (KE) from Coulomb repulsion. These low momentum features, and their temporal characteristics (their appearance and rise times), thus encode information on the UV-induced dissociation processes from which they are derived.

The XUV core-ionization probe method has some aspects in common with the ultrafast REMPI probe method noted previously.^10,22,23^ In the former case, the neutral dissociation can be ‘clocked’ and characterized once the inter-fragment distance exceeds *d*_c_ and CT from the core-ionized atom is no longer possible. REMPI detection is also blind to the very earliest stages of the bond fission process. In this case, the C–X separation has to extend beyond the range of neutral bonding interactions in order that the resonance conditions for detecting the free fragment species are satisfied. REMPI probing (for atomic and small molecular species) typically offers higher quantum state specificity, but REMPI efficiencies are typically also quantum state dependent, which hampers the quantification of product branching ratios. XUV ionization affects all quantum states of a chosen atom simultaneously, which can lead to more complex, overlapping spectra but also offers more opportunities for determining branching ratios when spectral components can be resolved.

The work reported here exploits the site-selective characteristics of XUV induced ionization and the *d*_c_ dependence of the ensuing CT process to explore the UV induced dissociation of two photochemically ‘richer’ iodides, 2- and 3-iodothiophene (henceforth abbreviated as 2IT and 3IT, respectively) which are depicted in Fig. 1. The experiment reports time-dependent changes in the momenta and yields of the resulting I^*n*+^ fragments, which are analysed to provide detailed information about competing dissociation processes in both molecules, and how these vary with the position of the iodine atom on the ring.

The sulfur-containing heterocycle, thiophene, is a key molecular sub-unit for organic electronics and functional materials.^24–26^ Thiophene derivatives, oligomers and polymers find use in organic solar cells,^27^ as photoswitches^28,29^ and as biological labels.^30^ Experimental and theoretical studies of bare thiophene associate the strong UV absorption peaking at wavelengths ∼230 nm with a π* ← π electron promotion and have identified both C–S bond extension and ring-puckering pathways for ππ* state population to access conical intersections with the ground state potential energy surface (PES) and form highly vibrationally excited ground state species (both the ring-closed parent and acyclic isomers).^31–36^ Studies of the derivatised counterpart thiophenone, where one of the carbon atoms is replaced by a carbonyl group, at the lower energy end of its UV absorption spectrum, have also shown that C–S bond extension and ring-opening is the dominant relaxation pathway that funnels photoexcited population back to the ground electronic state.^37,38^

Adding a halogen atom (X) to the ring, as in iodothiophene, introduces new relaxation pathways enabled by excited states formed by promoting an electron from an occupied n or π orbital to a σ* orbital localised on the C–X bond – henceforth described generically as (n/π)σ* states. Photoexcitation may populate such states directly (as in the alkyl halides) or indirectly, by non-adiabatic coupling from the more strongly absorbing ππ* excited states. In either case, photoexcitation can be expected to lead to efficient C–X bond fission that will compete with the non-adiabatic coupling to the ground state.^39–41^ Resonance Raman spectra recorded following photoexcitation of 2IT in cyclohexane (a weakly interacting solvent) at 245.9 nm and 252.7 nm showed activity in the C–I stretch mode (consistent with the initial stages of C–I bond fission) but also in several skeletal vibrational modes associated with C–S bond extension and possible ring-opening.^42^

The UV photolysis of 2IT has been investigated previously at many wavelengths in the range 220–305 nm using nanosecond laser REMPI in conjunction with VMI detection methods, as has the photolysis of 2-bromothiophene over a more limited wavelength range.^39^ The 2IT measurements yielded both I and I* products, both of which showed two-component total kinetic energy release (TKER) distributions. One, which is dominant at longer excitation wavelengths, peaks at TKER values approaching the maximum allowed by energy conservation, *i.e.* relatively little energy is deposited into internal modes of the C_4_H_3_S co-product. These products display anisotropic recoil velocity distributions, described by near-limiting *β* parameter values of ∼1.7,^39^ consistent with prompt dissociation following excitation of a transition whose dipole moment is aligned parallel to the C–I bond. Such behavior is in keeping with that observed following excitation to the dominant ^3^Q_0_(nσ*) state within the A-band of methyl iodide.^6,10^

The second component, which is centred at much lower TKER values, displays an isotropic angular distribution (*β* ~ 0) and gains in relative importance upon tuning to shorter excitation wavelengths where π*←π absorption is dominant. Complementary *ab initio* electronic structure calculations suggested that the slow I/I* products observed at shorter excitation wavelengths could arise *via* C–X bond fission from molecules that had decayed to vibrationally “hot” levels of the electronic ground state. Coupling to the ground state was again suggested to occur *via* elongation of a C–S bond, but whether the “hot” ground state molecules are mostly ring-closed or acyclic at the moment of C–I bond cleavage remained an open question.^39^

Ultrafast time-resolved studies of 2IT following 268 nm photoexcitation with transient absorption spectroscopy at wavelengths around the I 4d edge have been reported recently, along with supporting time-dependent density functional theory (TD-DFT) calculations.^41^ This experiment monitored the appearance of features attributable to atomic iodine as the C–I separation increased, returning an appearance time of 160 ± 30 fs, but identified no evidence of ring opening nor of the lower-TKER product pathway observed in the earlier nanosecond laser experiments. The supporting TD-DFT calculations suggested negligible cross-section for excitation to (n/π)σ* states and that the photophysics at this wavelength was dominated by initial π* ← π excitation followed by efficient non-adiabatic coupling to the dissociative (n/π)σ* PESs.

The present study employs site-selective ionization at the I 4d edge and the spatial dependence of CT to provide time and momentum-resolved data for the photodissociation dynamics of neutral 2IT and 3IT molecules following excitation at 262 nm, monitored *via* the I^2+^ signal. The time and momentum resolution afforded by the present experiments allows determination of appearance times of the high and low TKER products identified in the previous nanosecond laser studies, both of which arise on a picosecond (or faster) timescale. These data, considered along with the respective parent absorption spectra and prior electronic structure calculations,^39,41^ suggest an alternative rationale for the ‘high’ and ‘low’ TKER products observed at these relatively long wavelengths and reveal the extent to which these dynamics are affected by the position of the iodine atom on the ring.

## II. Experimental methods

The experiment was performed at the eXtreme UltraViolet (XUV) beamline (BL1) of Spring-8 Angstrom Compact free electron LAser (SACLA).^43^ The experimental apparatus is broadly similar to that outlined in ref. 44. XUV pulses at a photon energy of 95 eV (13.1 nm wavelength) were produced at a repetition rate of 60 Hz, with an estimated duration of ∼30 fs^45^ and were attenuated with a 0.5 μm thick Zr filter, prior to focusing to a spot size of ∼10 μm (1/*e*^2^) at the interaction point of the employed ion spectrometer. The shot-to-shot XUV pulse energies were measured upstream of the experiment using a gas intensity monitor,^46^ with a mean value of ∼30 μJ.^43^ Accounting for expected transmission of the beamline (∼90%) and the solid filter (∼19%), we estimate an on-target pulse energy of 5.1 μJ and a peak intensity of ∼4.4 × 10^14^ W cm^−2^.

The UV pump pulses (261.5 nm) were generated by frequency tripling the output of the BL1 optical laser system, which comprised a Ti:sapphire oscillator (Vitara, Coherent Inc.), a chirped-pulse regenerative amplification system (Legend Elite, Coherent Inc) and a home-built multipass amplifier.^43^ Prior to frequency tripling, the fundamental was attenuated by a variable neutral density filter to give UV pulse energies of ∼2 μJ. The UV pulses were focused into the interaction region with a lens (*f* = 2 m). The incoming UV laser beam was overlapped with the FEL output in a near-collinear geometry using a right-angle prism mirror. The delay between the optical laser and X-ray FEL (XFEL) pulse was scanned using a motorized delay stage. On a single shot basis, the jitter between the two pulses was measured using an arrival time monitor,^47^ and ultimately the data were rebinned following correction of this jitter.

The VMI spectrometer used in the experiment has been described in detail previously.^48^ Room temperature 2IT or 3IT molecules were expanded as an unseeded molecular beam through a pulsed General Valve. The beam was skimmed en route to the main spectrometer chamber, where it was intersected by the laser and XFEL pulses at a crossing angle of ∼45 degrees. Generated ions were accelerated by a series of electrodes under VMI conditions^49^ onto a time- and position-sensitive detector consisting of dual MicroChannel Plates (MCPs) and a hexanode delay-line, where their arrival time (*t*) and hit positions (*x*, *y*) were recorded. The calibration from detector coordinates to initial 3D ion momentum were performed using ion trajectory simulations. The TKER values presented in this paper were calculated assuming a two-body dissociation process forming the atomic iodine and thiophenyl (*m* = 83 amu) co-fragment.

The data were recorded for a range of UV – XUV delays ranging from approximately −2 to 6 ps in variable step sizes, where negative delays are defined as the XUV pulse preceding the UV pulse. The temporal overlap of the pulses was estimated from the depletion of I^2+^ yield arising from XUV induced Coulomb explosion of the ground state molecule. Fitting this signal as a function of time to an error function provides an upper limit estimate on the instrument response function of 175 fs. Following correction for XFEL timing jitter, the data were sorted by pump–probe delay into 50 fs bins in the range from −2 to 2 ps and into 200 fs bins from 2 to 6 ps. The total signal per pump–probe delay bin was normalized by the number of summed FEL shots.

## III. Results and discussion

### Pump–probe measurements

**Fig. 2:**
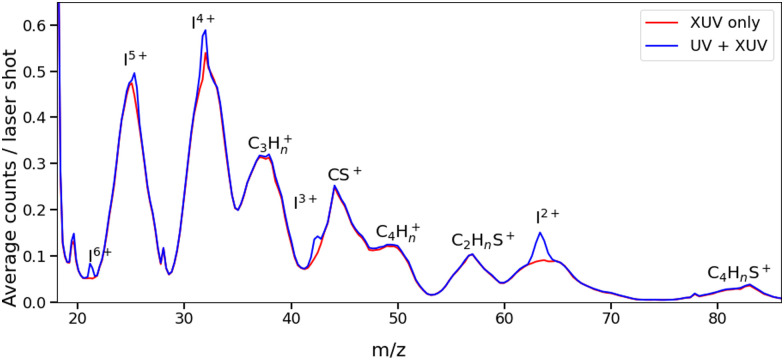
Mass spectra obtained from ionization of 2IT by UV + XUV pulses (blue) and by XUV pulses only (red). Each spectrum has been normalized by the total number of shots. The UV + XUV signal has been summed over all positive pump–probe delays (*i.e.* with the UV pulse preceding the XUV pulse).

Fig. 2 presents two mass spectra, obtained following ionization of 2IT by the XUV pulse alone (red), and when the XUV pulse was preceded by the UV pump pulse (blue). In both spectra, peaks associated with atomic iodine in charge states up to I^6+^ are observed, along with those associated with various carbon and/or sulfur-containing fragments. The full range of the mass spectrum, including the parent ion, and equivalent measurements for 3IT as well as the mass spectra obtained with the UV pump pulse alone are presented in the ESI.† The broad peaks observed in the mass spectra are due to fragments formed with a wide range of momenta following ionization and Coulomb explosion. The spectrum obtained from the combined effect of the UV and XUV pulses, which is averaged over all positive pump–probe delays, highlights that the main changes induced by the pump pulse are localised to the iodine fragment ions. The UV pump results in an increased intensity of the I^*n*+^ peaks over a very narrow *m*/*z* range, indicating that these fragments are released with a much lower spread of momenta. This effect is particularly obvious in the case of the I^2+^ ions, as these are generated with the highest yield and appear in the least congested region of the mass spectrum. Consequently, the subsequent figures presented in this paper are derived from an analysis of I^2+^ fragment signals. Other I^*n*+^ states have also been examined, and their delay-dependent momentum distributions exhibit qualitatively similar behaviour, albeit with a reduced signal-to-noise ratio, as presented in the ESI.†

**Fig. 3:**
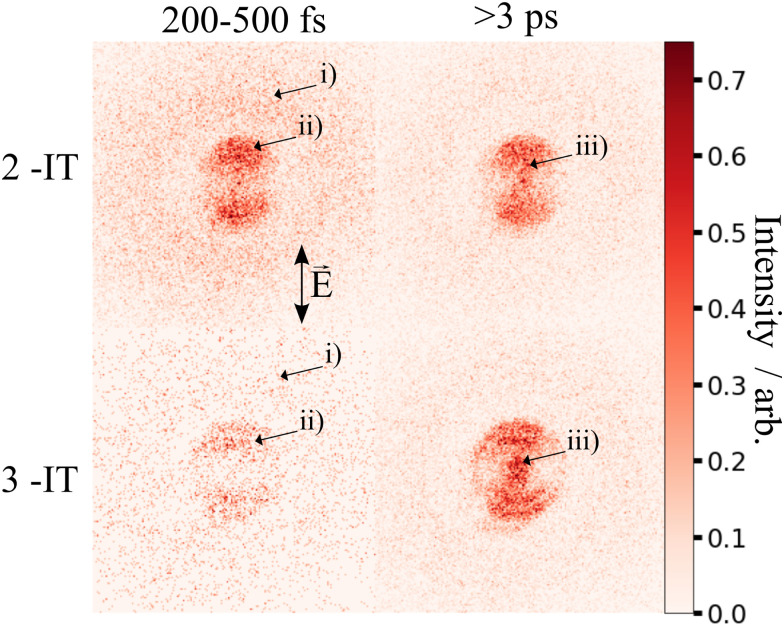
I^2+^ ion images following UV pump and subsequent XUV ionization of 2IT (top row) and 3IT (bottom row) summed over 200–500 fs (left column) and at all delays >3 ps (right column), representative of early and late dynamics. Each image was normalized to its peak intensity and the laser polarization axis *E* is marked in the top left panel.

The change in momentum of the I^2+^ fragments is clearly visible in the images collected as a function of pump–probe time delay. Fig. 3 shows I^2+^ fragment ion images from each isomer obtained at early (200–500 fs) and late (>3 ps) pump–probe delays. In these images, a higher radius corresponds to a higher fragment recoil velocity and thus to a higher momentum. We define the following three distinct features evident in the images from both isomers at progressively lower momentum: region (i) a weak, isotropic, and diffuse ring at large radius that is present at both positive and negative delays; region (ii) an anisotropic and intense ring at intermediate radius that appears at early positive delays and then persists; and region (iii) an isotropic ring at very low radius that only becomes apparent at late pump–probe delays. The angular variations of the signals in regions (ii) and (iii) were characterized by fitting a central slice through the momentum distribution to a second order Legendre polynomial, yielding *β* values of ∼1.4 and ∼1.0 for the region (ii) feature associated with photolysis of 2IT and 3IT, respectively, and *β* ∼ 0 for feature (iii) in both cases. These values indicate that the recoil velocity distribution of the I^2+^ fragments appearing in region (iii) is isotropic, but that the recoil of the fragments associated with region (ii) is strongly aligned parallel to the UV laser polarization axis. This is consistent with the previously observed result using ns REMPI.^39^ It is worth noting that more structure is visible in region (ii) of the images acquired using 3IT as opposed to 2IT, this is discussed in more detail later.

Region (i) is assigned to XUV-induced Coulomb explosion of the molecule following multiple ionization of the iodine atom. Ionization at the iodine site is followed by a redistribution of charge across the molecule and subsequent Coulomb explosion. The fragments created through this process have a broad range of momenta due to the large number of potential charges and explosion co-fragments that can be formed. Regions (ii) and (iii) correspond to significantly lower momenta, indicating that these signals do not originate from a Coulomb explosion process. The characteristics of these signals mimic those observed in previous experiments investigating CT processes in photodissociating molecules.^18,19^ As noted in the introduction, the onset of the low momentum peaks indicates the time at which charge on the ionised iodine atom can no longer transfer to the rest of the neutral nuclear framework. The critical distance, *d*_c_, at which such CT ceases marks the boundary between parent ions that will undergo Coulomb explosion, when CT is possible, and those that will not. Once the C–I bond length (in the present case) has passed this critical distance, the momenta of the measured I^2+^ fragment ions relate to the neutral dissociation dynamics, not the Coulomb interactions associated with multiple positively charged ions. The fact that two distinct momentum distributions are observed, with different associated formation timescales, implies two distinct neutral dissociation pathways – associated with regions (ii) and (iii), respectively.

**Fig. 4:**
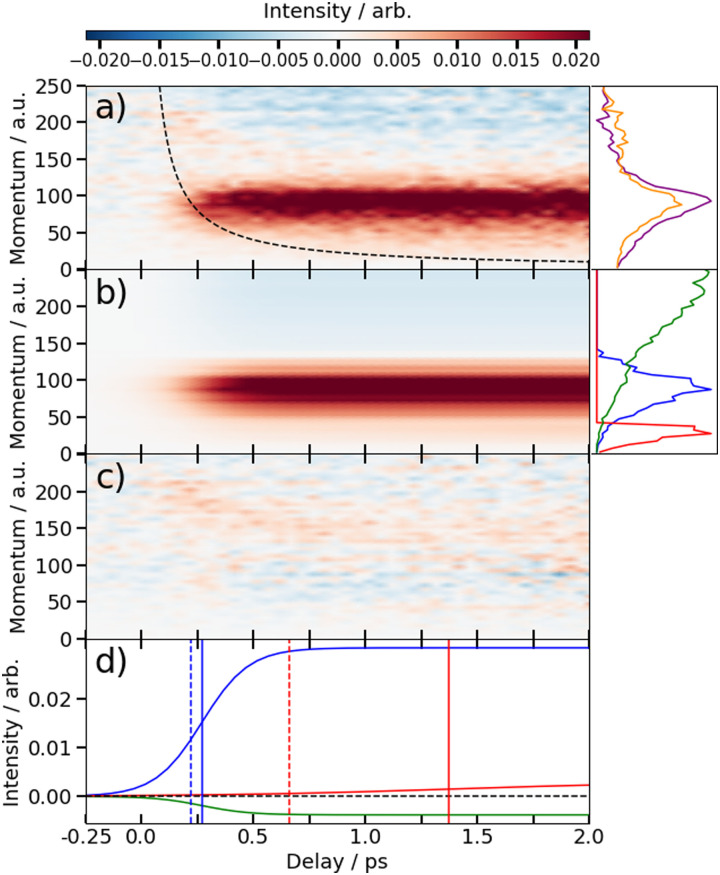
(a) Time-resolved difference map of the momentum of the I^2+^ fragments formed by 262 nm photolysis of 2IT and subsequent XUV probing (*i.e.* after subtracting the ground state (UV late) contribution). Measured in atomic units (a.u.). The intensity *vs.* momentum distributions at early (200–500 fs) and late (>3 ps) pump–probe delays are shown at the far right (plotted as orange and purple lines, respectively. The black dashed line depicts the expected appearance time of the I^2+^ fragments based on the OTB model as described in text. (b) Fit to the experimental data using a time-varying sum of the three intensity *vs.* momentum basis functions shown at the far right, as described in the text. The three basis functions are displayed with a common peak intensity. (c) Residual error between the experimental and fitted data. (d) Time-dependent amplitudes of the basis functions obtained from the fit. The green, blue and red curves are associated with ground state, prompt and delayed dissociation basis functions respectively. Dashed vertical lines in panel (d) mark the expected appearance time based on the OTB model and the solid vertical lines mark the appearance time obtained in the fit.

**Fig. 5:**
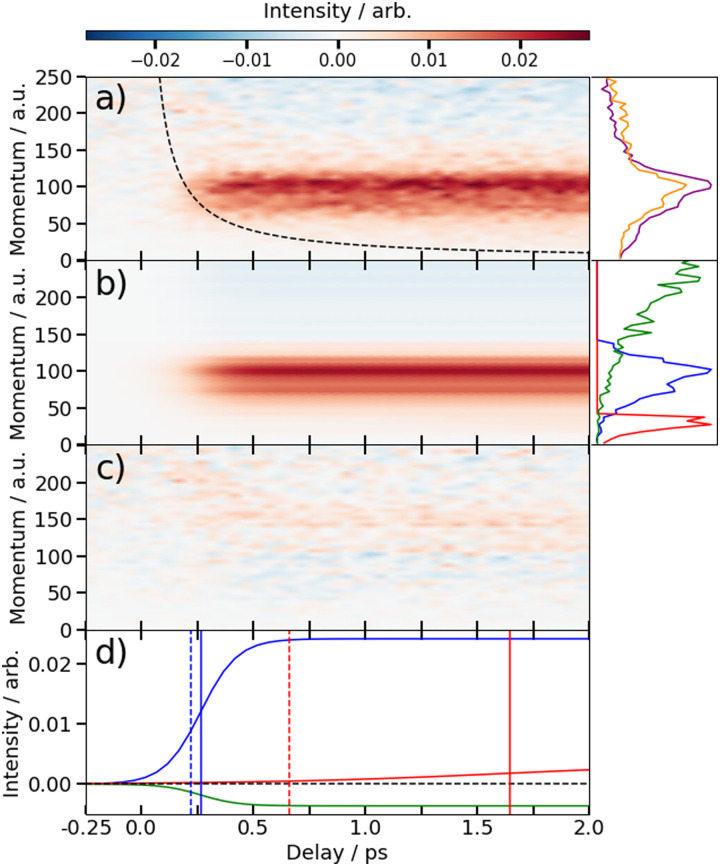
(a) Time-resolved difference map of the momentum of the I^2+^ fragments formed by 262 nm photolysis of 3IT and subsequent XUV probing (*i.e.* after subtracting the ground state (UV late) contribution). Measured in atomic units (a.u.). The intensity *vs.* momentum distributions at early (200–500 fs) and late (>3 ps) pump–probe delays are shown at the far right (plotted as orange and purple lines, respectively. The black dashed line depicts the expected appearance time of the I^2+^ fragments based on the OTB model as described in text. (b) Fit to the experimental data using a time-varying sum of the three intensity *vs.* momentum basis functions shown at the far right, as described in the text. The three basis functions are displayed with a common peak intensity. (c) Residual error between the experimental and fitted data. (d) Time-dependent amplitudes of the basis functions obtained from the fit. The green, blue and red curves are associated with ground state, prompt and delayed dissociation basis functions respectively. Dashed vertical lines in panel (d) mark the expected appearance time based on the OTB model and the solid vertical lines mark the appearance time obtained in the fit.

The time-dependent signals shown in Fig. 3 can be resolved more clearly by plotting the momentum distributions (after subtracting the UV late (*i.e.* ground state) signal) as a function of pump–probe delay. Panel (a) of Fig. 4 and 5 summarise the momenta (in atomic units) of the I^2+^ fragments produced from UV photolysis of 2IT and 3IT, respectively. In each figure, red indicates an increase in intensity with respect to the (UV late) parent ground state signal, while blue represents a depletion. After time-zero, the depletion of the ground state contribution to the signal is obvious as a broad area of blue at high momentum (>120 a.u.), while the products of UV photodissociation are revealed through the strong enhancements at low (<120 a.u.) momentum values.

The average momentum distribution obtained at early (200–500 fs) and late (>3 ps) delay times are projected on the right-hand side of panel (a) in Fig. 4 and 5. The projections illustrate the two distinct, albeit significantly overlapped, pump–probe features of this spectrum as highlighted in the images of Fig. 3. The projections show an intense peak centered at ≈90 a.u. with an appearance time close to time-zero. The intensity of this main peak continues to increase, but a second, weaker, peak centered at ≈30 a.u. is now also apparent in the late time spectrum. Both features appear after time-zero, and the associated momentum distributions peak away from zero momentum and are independent of the pump–probe delay. As this manuscript focuses on the fitting procedure described in subsequent paragraphs these features have been plotted as integrated transients in the ESI,† for clarity. These momentum distributions are attributable to 262 nm photoinduced C–I bond cleavage of the neutral molecule. Their constancy (in time) indicates minimal contribution from any Coulomb repulsion, implying that these signals are only observable once the breaking C–I bond length exceeds *d*_c_. We note that the value of *d*_c_ is charge state-dependent and will be larger for higher charge states. This is shown in the ESI,† where we plot equivalent data for the I^3+^ and I^4+^ charge states.

Note, the maps presented in panel (a) of Fig. 4 and 5 do also show features attributable to molecules undergoing Coulomb explosion while in the act of dissociating. The transient enhancements visible at high momentum (at early times), that progressively decline as the pump–probe delay increases arise from the small (but non-zero) XUV ionization cross-section of the partner fragment. The time-delayed XUV pulse thus produces some such singly charged fragments in tandem with I^2+^ species. Their mutual repulsion decreases with increasing pump–probe delay, as the two fragments move away from one another, leading to the characteristic Coulomb curve.^50^ The onset of the Coulomb curve is used to define the experimental time-zero as outlined in the ESI.†

### Kinetics of the neutral dissociation process

Information on the timescales associated with the neutral dissociation processes were obtained using an adapted global fitting procedure. Ignoring the weak contribution from the Coulomb curve, the full momentum distribution can be viewed as a composite of three basis spectra representing, (i) XUV-induced Coulomb explosion of the ground state molecule yielding signal at high momentum, (ii) prompt UV-induced dissociation signal at intermediate momentum, and (iii) delayed UV-induced dissociation signal at low momentum. All of these components overlap but, based on their different temporal properties, the necessary basis spectra can be derived from the experimental data. Pre-time-zero, the measured signal is solely from XUV-induced Coulomb explosion of the ground state molecule. The average pre-time-zero spectrum is thus used as the first basis function. The low momentum part of this XUV-induced Coulomb explosion distribution is shown by the green curve at the right of panel (b) in Fig. 4 and 5. At early positive pump–probe delay times (200–500 fs), the measured spectrum is composed of the depleted ground state signal and the prompt dissociation signal. To obtain an isolated spectrum associated with the prompt dissociation signal, a scaled subtraction of the ground state spectrum was performed. Such scaling was necessary, as these two signals overlap and subtracting the full pre-time-zero signal would result in a double weighting of the ground state contribution in the overlap region. This can be avoided by scaling the ground state contribution such that the average signal is zero in regions where only depletion is observed. The ‘prompt’ dissociation basis functions obtained in this way are shown as the blue lines on the right-hand side of panel (b) in the respective figures. A similar procedure, wherein the early time spectrum was subtracted from the late time spectrum (delays >3 ps) yielded the ‘delayed’ dissociation basis functions shown in red at the right-hand side of panel (b) in the respective figures.

The observed changes in the intensities of the basis functions correlate with the time at which the I^2+^ species is sufficiently separated from the rest of the molecule that CT can no longer occur. These onsets mark the time at which enhancements of the low momentum signal associated with neutral dissociation start to contribute and the time at which the XUV-induced Coulomb explosion signal depletes. Since the prompt dissociation is the major process, the time at which CT stops will lead to a depletion of the ground state Coulomb explosion signal, and the onset of the prompt neutral dissociation signal. Thus, the same temporal characteristics (but with opposite sign) are ascribed to these two processes. The weaker, delayed dissociation signal is then characterised by a different set of temporal characteristics. To extract quantitative information on the temporal characteristics of the signals the momentum maps were fit to a weighted sum of logistic functions given by eqn (2):2
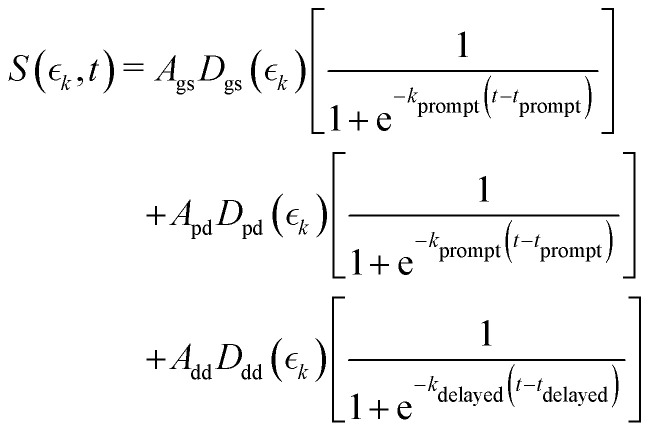
where 
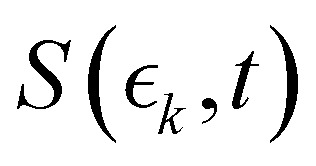
 is the signal intensity dependent on the pump–probe delay time, *t*, and momentum, 
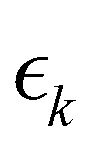
, dependent signal intensity. The momentum-dependent basis functions obtained from the experiment (plotted at the far right of panel b) in Fig. 4 and 5) are given by 
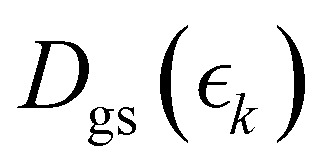
, 
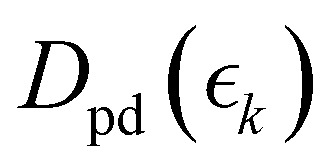
 and 
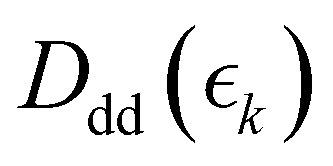
 for the ground state depletion, prompt dissociation and delayed dissociation signals, respectively. We provide expanded versions of the basis function plots in the ESI.† The centre of the rise of the logistic functions in time are defined by, *t*_prompt_, for the ground state depletion and prompt dissociation signal, and *t*_delayed_ for the delayed signal. *k*_prompt_ and *k*_delayed_ are the rate constants associated with the prompt and delayed signals, respectively. The amplitude of each term in the sum is then given by the constant, *A*_*i*_, which controls the relative contributions of that basis function to the overall signal.

The values for *k*_prompt_, *k*_delayed_, *t*_prompt_, *t*_delayed_ and *A*_gs_, *A*_pd_ and *A*_dd_ for the 2IT and 3IT data were optimized *via* least squares fitting. The optimized fit parameters are given in Table 1 with the calculated signal and residual maps for 2IT and 3IT given in panels (b) and (c) of Fig. 4 and 5, respectively. This simple model provides an excellent fit to the overall intensity profile associated with the neutral dissociation signal, and the contribution from the respective Coulomb curves are more obvious in the residual plots. Panel (d) in each figure shows the time-dependent contribution of each component, highlighting the delayed appearance of the low momentum feature and its much slower rate of signal increase. A further comparison between the transients of experimental and simulated data is presented in the ESI.†

**Table 1:** Values of *k*_*i*_, and *t*_*i*_ obtained from fitting the experimental data to eqn (2), and the prompt/delayed dissociation branching ratios (BR) obtained from the fit amplitudes

	*k*_prompt_/ps^−1^	*k*_delayed_/ps^−1^	*t*_prompt_/fs	*t*_delayed_/ps	BR
2IT	10 ± 0.4	2.0 ± 0.9	267 ± 50	1.4 ± 0.1	23 ± 2
3IT	12 ± 1	1.9 ± 0.7	264 ± 50	1.6 ± 0.1	16 ± 3

At this point we reiterate that the observation of the UV-induced I^2+^ signal occurs once the C–I seperation has reached a critical distance, *d*_c_, beyond which CT can no longer occur. *d*_c_ can be calculated using the classical OTB model and assuming that the neutral molecules dissociate promptly to an I atom and the thiophenyl radical partner. These calculations assume an ionization potential of 8.0 eV for the thiophenyl radical based on the Hess' law calculation outlined in the ESI.† The 8.0 eV ionization potential gives a *d*_c_ value for the I^2+^ ion and neutral thiophenyl radical of 6.89 Å. Assuming prompt C–I bond fission and that the asymptotic recoil velocity is reached instantaneously allows estimation of the time required to reach *d*_c_. The approximation that the asymptotic velocity is reached instantly will impact the very early time signal, but this velocity is likely reached within a few tens of femtoseconds assuming ballistic motion along a repulsive potential^15,51^ and the predicted time should lie within the present margins of error and have little consequence on the conclusions drawn from this analysis. The results of this calculation of the time taken to reach *d*_c_ assuming a constant separation velocity are overlayed as a dashed black line in panel (a) of Fig. 4 and 5.

The values obtained from the OTB model can be compared with appearance times obtained from the fit. Given the spread of momenta associated with each channel, the time returned from the fit correspond to the average times taken to reach *d*_c_. The observation times for the ‘prompt’ dissociation from both isomers are ∼265 fs (Table 1), shown by a solid blue line in panels d) of Fig. 4 and 5. The associated momentum distributions peak at ∼90 a.u., which implies an expected appearance time of 220 fs as marked by a dashed blue line in panel (d) of Fig. 4 and 5. Given the approximation that the asymptotic velocity is reached instantly, the agreement between these values is well within our expected error bounds, and is fully consistent with the idea that the higher momentum fragments arise *via* a ‘prompt’ or direct photodissociation process. Essentially perfect agreement can be achieved with the OTB model by assuming an asymptotic dissociation momentum of 75 a.u., a value close to the centres of the respective skewed momentum distributions shown to the right in panels (b) of Fig. 4 and 5.

As Table 1 shows, the observation times for the delayed signals are ∼1.4 ps and ∼1.6 ps for the 2IT and 3IT isomers, respectively, shown by a solid red line in panel (d) of Fig. 4 and 5. The peak of the delayed momentum distributions in each case is ∼30 a.u. which, if these signals also arose *via* a prompt dissociation, would lead to an OTB model predicted appearance time of ∼660 fs, shown by the dashed red line in panel (d) of Fig. 4 and 5. The ∼1 ps between these two values is most readily explained by invoking a delayed dissociation process, a point we will return to later in this Section.

The rate constants in Table 1, which define the rate at which the signals rise, give associated time constants (1/*k*) of 100 fs and 80 fs for the prompt dissociation products from the 2IT and 3IT isomers, respectively. The main factor determining the observed rate of signal growth is the range of velocities contained within the associated distributions. The momentum distributions shown in blue in panels (b) of Fig. 4 and 5 have a full width at half maximum (FWHM) spanning the range 67–110 a.u. In the OTB model, the steep gradient seen in the appearance times over this range of momenta leads to a narrow range of expected appearance times. Assuming prompt C–I bond fission, the measured momentum distributions would translate into a spread of appearance times of ∼120 fs, similar to, but slightly longer than, the respective 1/*k* values. The difference could be a consequence of the Coulomb curve on the high momentum side of the distribution which causes an additional (and, from the perspective of the neutral dissociation process, artificial) broadening.

Similar analyses for the delayed signal feature return spreads of appearance times at *d*_c_ of ∼770 fs for 2IT and ∼620 fs for 3IT (from their respective FWHM momentum ranges of 17–50 a.u. and 18–41 a.u.), in good accord with the longer time constants returned by the fit (Table 1). This reinforces the conclusion that the rate constants returned by the fit are dominated by the spread of recoil velocities, that the time offsets report on the average time taken to reach *d*_c_, and that the kinetic parameters obtained for the two isomers are very similar.

The fit also allows estimation of the relative contributions of the prompt and delayed dissociation signals. By taking the amplitudes, *A*_*i*_, obtained from the fits and multiplying these by the basis functions, we can integrate the resultant signal to obtain the asymptotic channel dependent intensity. The ratio of these intensities yields a branching ratio, BR in Table 1. This analysis shows that the prompt signal makes the dominant contribution to the total yield following excitation of both isomers at 262 nm and that the delayed component has relatively greater importance in the 3IT isomer. The previous I and I* product-resolved, nanosecond VMI studies of 2IT photolysis in this wavelength region also suggested a dominant role for the direct dissociation pathway.^39^ BRs for the two pathways were not determined in that work but, given the basis functions derived in the present work, the earlier data suggest BR values closer to ∼4 : 1. Several factors may contribute to this apparent difference. As noted above, the intensity of direct dissociation products is likely to be inflated on its high momentum side by a contribution from the Coulomb curve converging to the same asymptotic momentum limit. Conversely, the present analysis may well underestimate the relative yield of the delayed, low momentum, products. In the present experiments, the intensities are calculated based on the basis functions and intensities fit over the 6 ps time range measured. The nanosecond VMI studies employed orders of magnitude longer pump–probe time delays (20–45 ns). If there is any probability for internal conversion of photoexcited molecules by coupling back to the ground state following excitation at 262 nm, it is plausible that the resulting vibrationally ‘hot’ ground state species might dissociate to yield additional slow I atoms over this much longer time scale. Such delayed contributions would not be visible on the few ps timescale measured here.

### Energy partitioning in the fragments

**Fig. 6:**
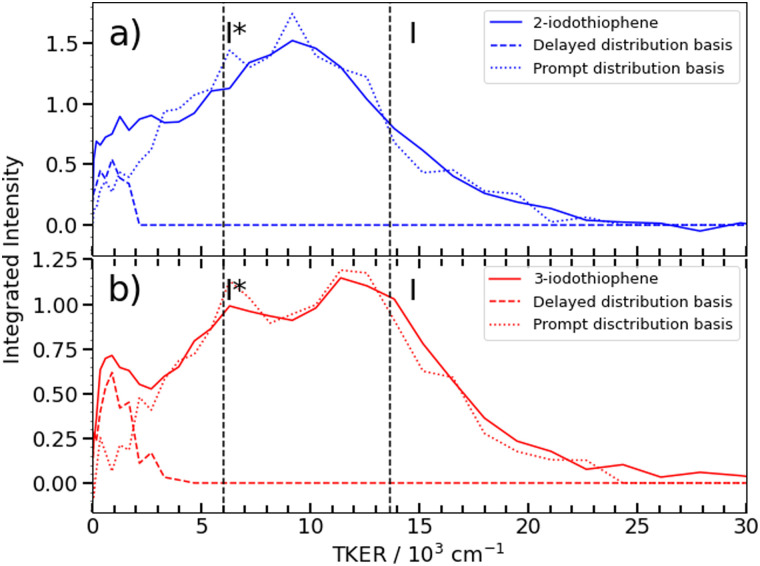
TKER distributions obtained from the I^2+^ asymptotic velocity distributions from 262 nm photolysis of (a) 2IT (blue) and (b) 3IT (red), assuming two-body dissociation into I + C_4_H_3_S. The dashed curves show the TKER distributions associated with the ‘prompt’ and ‘delayed’ dissociation channels (derived from the respective momentum basis functions), while the dashed vertical lines show the maximum TKER values associated with forming I and I* photoproducts, given *D*_0_(R−I) = 24 500 cm^−1^.

Fig. 6 shows asymptotic TKER distributions obtained for the dissociation products from the two isomers, derived from the pump–probe data taken at *t* > 3 ps and assuming a mass *m* = 83 amu for the partner fragment. The reported C–I bond strength in 2IT, *D*_0_(R−I) = 24 500 cm^−1^, implies a maximal TKER (TKER_(max)_) value shown by the right hand dashed vertical line in Fig. 6(a). The observation of some signal with TKER > TKER_(max)_ is attributed to a combination of finite experimental resolution and the aforementioned contribution from the Coulomb curve. The corresponding TKER_(max)_ value for forming spin–orbit excited I* fragments (with *E*_so_ = 7603 cm^−1^) is also shown. The C–I bond strength in 3IT has not been reported, but previous VMI studies of the Br and Br* atoms arising from 267 nm photolysis of 2BrT and 3BrT assumed identical C–Br bond strengths,^40^ and recent threshold photoelectron imaging studies of the furanyl anion (furan is the oxygen analogue of bare thiophene) deduced essentially identical alpha- and beta-C–H bond strengths.^52^ Henceforth, identical *D*_0_(R−I) values for 2IT and 3IT are assumed.

Fig. 6(b) shows that the TKER distribution following 262 nm photolysis of 3IT peaks ∼2500 cm^−1^ higher in energy *cf.* that from 2IT. Relative to 2IT, the higher TKER component from 3IT photolysis also appears sharper, revealing two maxima with a spacing consistent with formation of both I and I* products. No such splitting is evident in the 2IT data, though previous studies have revealed formation of both I and I* photoproducts when exciting 2IT at similar wavelengths. This difference could be accommodated by assuming that the C_4_H_3_S partners in the photodissociation of the 2IT isomer are formed in a broader spread of internal energy states. The present data for both isomers reveal a strong propensity for channelling the UV photon energy in excess of that required for C–I bond fission into product translation, but also show relatively greater internal excitation of the partner fragment in the case of 2IT photolysis.

### Excited state photochemistry and the dissociation dynamics

**Fig. 7:**
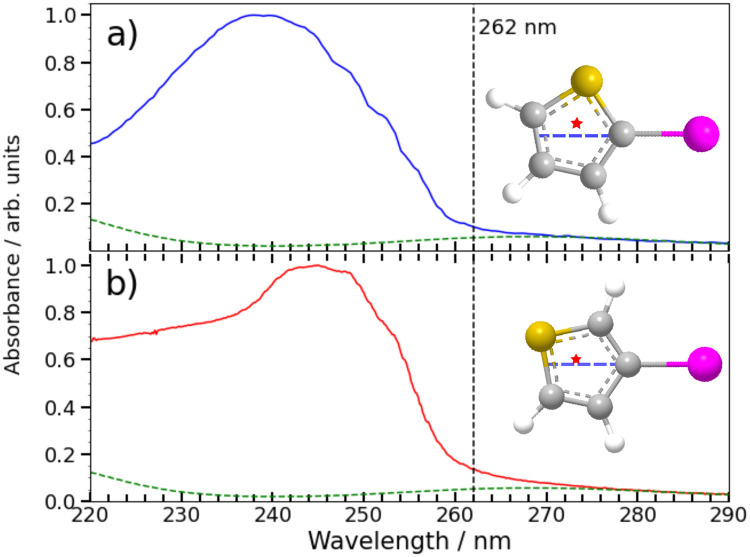
Gas phase absorption spectrum of 2IT (a) and 3IT (b) are plotted as solid lines along with a scaled absorption spectrum of CH_2_BrI (dashed line) to emphasise the weak long wavelength absorption attributable to nσ* excitation in both 2- and 3-iodothiophene. The intensity scale has been normalized to the maximum measured for each isomer to show relative changes in the shape. The structure of the two isomers are plotted as insets with the C–I bond vector highlighted as a dashed blue line and the center of mass of the thiophenyl radical marked by a star.

Any detailed description of the dissociation dynamics requires knowledge of the number and nature of excited states contributing to the parent absorption at the wavelength of interest. Fig. 7 shows room temperature absorption spectra of (a) 2IT and (b) 3IT at wavelengths between 220–290 nm measured using the method outlined in the ESI.† Each spectrum has been normalized to their respective maximum absorbance. The spectrum of 2IT agrees well with that reported in an earlier study that also highlighted the number of excited states with vertical excitation energies in the relevant range.^39^ The spectra of the two isomers have many similarities, and both can be understood as a combination of two distinct types of excitation, to (i) one or more dissociative (n/π)σ* states localized on the C–I bond, reminiscent of the A band absorptions of the alkyl iodides;^1,8^ and (ii) a much more strongly absorbing, ring-centered, diabatically bound ππ* state, the PES for which favours ring expansion/distortion.^37,39^ To indicate the contribution of the (n/π)σ* states to the overall absorption spectrum, we plot a scaled version of the absorption spectrum of CH_2_BrI as a dashed line in Fig. 7(a) and (b). The spectrum of CH_2_BrI provides an illustrative absorption envelope for the (n/π)σ* states as seen in the A bands of many alkyl iodides, all of which comprise a continuous absorption spanning ∼40–50 nm (FWHM) with an approximately Gaussian profile.^1^ The difference between this and the iodothiophene spectra is then the contribution of the ππ* state. These figures serve to highlight that the partial cross-sections for the two families of excitation in 2IT and 3IT will be wavelength dependent and, at least mildly, isomer dependent.

The present and previous^39,41^ photodissociation data are now discussed within this framework. The illustrative CH_2_BrI spectrum shown in Fig. 7 suggest that most absorption at 262 nm is attributable to σ* ← (n/π) excitations. All such states are repulsive with respect to C–I bond extension, and analogy with the alkyl halides suggest that excitation to the so-called ^3^Q_0_ excited state will have the largest cross-section in this region. This state correlates with I* products, but efficient non-adiabatic coupling to other (n/π)σ* states provides a route to ground state I atom products. Consistent with these expectations, we note that the high momentum I and I* products appear promptly (consistent with direct excitation to a repulsive PES), show predominant parallel recoil anisotropy (consistent with excitation to the ^3^Q_0_ excited state), and that most of the photon energy in excess of that required for bond fission is released as product translational energy. All of these characteristics mimic the well-characterised alkyl halide photofragmentation dynamics. The (relatively) greater internal excitation of the radical fragment from 2IT photolysis may be attributable to increased product rotation. As insets to Fig. 7, we plot the structure of the two isomers, highlighting the C–I bond vector as a dashed blue line and marking the center of mass of the departing thiophenyl radical with a star. In 3IT the C–I bond vector lies closer to the centre of mass than in 2IT. From a simple ballistic picture, the impulse from the recoiling I atom would thus be expected to lead to higher rotational energy in the radical fragments from 2IT photolysis.

We note that such excitations, σ∗ ← (n/π), were discounted in the recent transient absorption spectroscopy study of 2IT photolysis, because the supporting (singlet state only) TD-DFT calculations predicted that they should have negligible cross-section.^41^ But these calculations also revealed a very efficient non-adiabatic coupling pathway from the ππ* state to the (n/π)σ* continua, which is very likely to be responsible for the ‘delayed’, low momentum products observed in the present work. π* ← π excitation initiates ring breathing motions, which are amplified during non-adiabatic coupling to the (n/π)σ* continua.^41^ The deduced ∼1 ps delay prior to accessing the dissociative states is qualitatively consistent with that predicted by the TD-DFT calculations, and the activation of nuclear motions orthogonal to the C–I bond dissociation coordinate (motions which carry through into the radical products) explains the observed lower product recoil velocities and momenta. Further, the finding that the basis functions used to describe the delayed dissociation products in Fig. 4(b) and 5(b) peak at finite momentum (not zero) is also consistent with eventual bond fission on a repulsive PES. Comparing the absorption spectra of 2IT and 3IT, the red shift of the absorption maximum in 3IT suggests an enhanced relative contribution from π* ← π absorption in the latter at wavelength ∼262 nm, consistent with the greater relative yields of ‘delayed’ dissociation products (*i.e.* the lower BR) observed for the 3IT isomer in the present work.

The analysis outlined above leads to several predictions. The partial cross-sections for σ* ← (n/π) and π* ← π absorption are both wavelength dependent. Excitation of 2IT and 3IT at wavelengths longer than 262 nm should yield even larger BRs by enhancements in the prompt dissociation channel. The photochemistry should be dominated by direct dissociation following excitation to one or more (n/π)σ* states, yielding translationally excited I and I* fragments with anisotropic recoil velocity distributions. Conversely, the partial cross-section for π* ← π excitation should progressively dominate upon tuning to shorter wavelengths. Based on the present analysis, this should boost the relative yield of the delayed C–I bond fission channel enabled by non-adiabatic coupling to the (n/π)σ* continua, and the formation of less translationally excited I and I* products. This predicted energy disposal is broadly consistent with the imaging data for the (dominant) ground state I atoms in the earlier nanosecond VMI studies of 2IT photolysis at shorter UV wavelengths.^39^ Finally, analogy with thiophene and thiophenone photochemistry^37,38^ has encouraged suggestions that π* ← π excitation in 2IT and 3IT could also enable a rival ring-opening channel, non-adiabatic coupling to the ground state, and subsequent dissociation of the resulting ‘hot’ cyclic or acyclic ground state molecules. The present experiments are blind to the bonding within the cofragment. Furthermore, a 262 nm photon provides insufficient energy to induce both C–S bond fission and a subsequent C–I bond fission without an H atom transfer within the initial biradical species.^39,41^ But, seeking evidence for a rival ring-opening process when exciting 2IT or 3IT at shorter wavelengths within the ππ* absorption band remains a worthwhile challenge.

## IV. Conclusion

The fragmentation dynamics of 2IT and 3IT following excitation at 262 nm have been investigated by time-resolved VMI methods following site-selective XUV ionization at the I 4d edge. Two C–I bond fission channels are identified in each case. The dominant process in both isomers at this wavelength is direct dissociation, yielding translationally excited I atom fragments with anisotropic recoil velocity distributions. The second, relatively much weaker, channel involves delayed formation of iodine fragments with an isotropic and much lower momentum distribution. Energy conservation requires that the radical fragment arising *via* this second channel carry a much higher level of internal excitation. The relative importance of the delayed channel is larger for the 3IT isomer when exciting at 262 nm.

The dominant process in both isomers is assigned to direct dissociation following excitation to one or more (n/π)σ* states. The 3IT photolysis data clearly show formation of translationally excited I and I* products, as found in the previous nanosecond imaging studies of 2IT at similar UV wavelengths.^39^ The ultrafast appearance time of these ‘prompt’ products agrees well with that determined in a recent transient XUV absorption study of 2IT photolysis at 268 nm,^41^ and is similar to values reported for C–I bond fission following excitation of various alkyl halides to their respective A band continua.^22,23^ The present findings confirm that 262 nm photoexcitation of both 2IT and 3IT populates one or more (n/π)σ* PESs directly, contrary to the conclusions reached in a recent ultrafast transient absorption spectroscopy study of 2IT photolysis.^41^ It is worth noting the XUV absorption study is not as sensitive to the appearance of the ‘delayed’ component which we are able to identify in our study. This is because the two channels are strongly spectroscopically overlapped, therefore the delayed dissociation signal would be overshadowed by the much stronger direct dissociation. Here, we are able to overcome this as it investigates the two very different kinetic energies of the fragments produced *via* the mechanisms. An alternative mechanism advocated in that study^41^ involving initial π* ← π excitation followed by efficient non-adiabatic coupling to the (n/π)σ* continua is deduced to be responsible for the observed minor ‘delayed’ dissociation channel. The relative importance of these two dissociation channels is predicted to vary markedly with excitation wavelength. The latter process is expected to dominate at shorter wavelengths, where the parent absorption is dominated by π* ← π absorption, but may be in competition with unimolecular decay of ‘hot’ ground state species formed by radiationless transfer driven by ring-opening or ring-puckering motions in the photoexcited parent (as have been identified in bare thiophene and in thiophenone). Clearly, further comparative ultrafast pump–probe studies of these isomers, and their brominated analogues, at a wider range of excitation wavelengths would be rewarding.

## Author contributions

R. F. conceived the experiment, the plan for which benefited from further input from F. A., M. A., J. H., R. M. and K. N. The sample delivery and spectrometer were prepared by J. H. and K. N. Y. K. arranged the data acquisition software and S. O. prepared the beamline. The experiment was conducted onsite by M. F., T. G., J. H., Y. H., A. H., H. I., Y. K., S. M., K. N., and A. N., with online participation in the experiment by all coauthors. The experimental data were analyzed by F. A., Y. K. and W. R. Finally, F. A., M. A., R. F., R. M. and W. R. interpreted the results and wrote the manuscript with input from all the authors.

## Conflicts of interest

There are no conflicts to declare.

## Supplementary Material

CP-026-D3CP06079A-s001
