# Supplementary material for: Exploring the ultrafast and isomer-dependent photodissociation of iodothiophenes via site-selective ionization
Source: Phys Chem Chem Phys. 2024 Apr 5;26(16):12725–37. doi: 10.1039/d3cp06079a (PMC11041866; doi:10.1039/d3cp06079a)
Supplement: CP-026-D3CP06079A-s001 [file CP-026-D3CP06079A-s001.pdf]

Electronic Supplementary Material (ESI) for Physical Chemistry Chemical Physics.
This journal is © the Owner Societies 2024

Supplementary Information: Exploring the Ultrafast and Isomer-Dependent Photodissociation of Iodothiophenes via Site-Selective Ionization

Weronika O. Razmus,[1] Felix Allum,[2,3,4] James Harries,[5] Yoshiaki Kumagai,[6] Kiyonobu Nagaya,[7] Surjendu Bhattacharyya,[8] Mathew Britton,[3] Mark Brouard,[2] Philip H. Bucksbaum,[3] Kieran Cheung,[2] Stuart W. Crane,[9] Mizuho Fushitani,[10] Ian Gabalski,[3] Tatsuo Gejo,[11] Aaron Ghrist,[3,4,12] David Heathcote,[2] Yasumasa Hikosaka,[13] Akiyoshi Hishikawa,[10,14] Paul Hockett,[15] Ellen Jones,[2] Edwin Kukk,[16] Hiroshi Iwayama,[17] Huynh V. S. Lam,[8] Joseph W. McManus,[2] Dennis Milsesevic,[2] Jochen Mikosch,[18] Shinichirou Minemoto,[19] Akinobu Niozu,[20] Andrew J. Orr-Ewing,[21] Shigeki Owada,[22,23] Daniel Rolles,[8] Artem Rudenko,[8] Dave Townsend,[9,9] Kiyoshi Ueda,[24,25] James Unwin,[2] Claire Vallance,[2] Anbu Venkatachalam,[8] Shin-ichi Wada,[20] Tiffany Walmsley,[2] Emily M. Warne,[2] Joanne Woodhouse,[1] Michael Burt,[2] Michael N. R. Ashfold,[21] Russell S. Minns,[1,*] and Ruaridh Forbes[4,†]

[1] *School of Chemistry, University of Southampton, Highfield, Southampton, SO17 1BJ, UK*
[2] *Chemistry Research Laboratory, Department of Chemistry, University of Oxford, Oxford OX1 3TA, UK*
[3] *PULSE Institute, SLAC National Accelerator Laboratory, 2575 Sand Hill Road, Menlo Park, CA 94025, USA*
[4] *Linac Coherent Light Source, SLAC National Accelerator Laboratory, 2575 Sand Hill Road, Menlo Park, CA 94025, USA*
[5] *QST, SPring-8, Kouto 1-1-1, Sayo, Hyogo, Japan*
[6] *Department of Applied Physics, Tokyo University of Agriculture and Technology, Tokyo, Japan*
[7] *Department of Physics, Kyoto University, Kyoto 606-8502, Japan*
[8] *J.R. Macdonald Laboratory, Department of Physics, Kansas State University, Manhattan, Kansas 66506, USA*
[9] *Institute of Photonics and Quantum Sciences, Heriot-Watt University, Edinburgh, EH14 4AS, United Kingdom*
[10] *Department of Chemistry, Graduate School of Science, Nagoya University, Furo-cho, Chikusa, Nagoya, Aichi 464-8602, Japan*
[11] *Graduate School of Material Science, University of Hyogo, Kuoto 3-2-1, Kamigori-cho, Ako-gun, Hyogo 678-1297, Japan*
[12] *Department of Applies Physics, Stanford University, Stanford, California 94305, USA*
[13] *Institute of Liberal Arts and Sciences, University of Toyama, Toyama 930-0194, Japan*
[14] *Research Center for Materials Science, Nagoya University, Furo-cho, Chikusa, Nagoya, Aichi 464-8602, Japan*
[15] *National Research Council of Canada, 100 Sussex Dr. Ottawa, ON K1A 0R6, Canada*
[16] *Department of Physics and Astronomy, University of Turku, FI-20014 Turku, Finland*
[17] *Institute for Molecular Science, Okazaki 444-8585, Japan*
[18] *Department of Physics, University of Kassel, Heinrich-Plett-Strasse 40, 34132 Kassell, Germany*
[19] *Department of Physics, Graduate School of Science, The University of Tokyo, 7-3-1 Hongo, Bunkyo-ku, Tokyo 113-0033, Japan*
[20] *Graduate School of Advanced Science and Engineering, Hiroshima University, Higashi-Hiroshima 739-8526, Japan*
[21] *School of Chemistry, University of Bristol, Cantock's Close, Bristol BS8 1TS, UK*
[22] *RIKEN SPring-8 Center, Sayo, Hyogo, 679-5148, Japan*
[23] *Japan Synchrotron Radiation Research Institute, Hyogo, Japan*
[24] *Department of Chemistry, Tohoku University, Sendai 980-8578, Japan*
[25] *Department of Condensed Matter Physics and Photon Science, School of Physical Science and Technology, ShanghaiTech University, Shanghai 201210, China*

[*] r.s.minns@soton.ac.uk
[†] ruforbes@stanford.edu

I. TIME-ZERO DETERMINATION

Time-zero, defined as the time of maximum overlap of the UV and XUV laser pulses, was estimated based on the time at which the Coulomb curve becomes visible in the experimental data. This was chosen as all other signals will have an intrinsic delay in their appearance due to their distance-dependence. The Coulomb signal is isolated in the time-resolved residual colormap visible in Figures 3 and 4 panel c) of the main manuscript. The onset of this feature will be impacted by molecular dynamics as well as the cross-correlation of the laser pulses, therefore it serves as the upper limit for both time-zero and cross-correlation FWHM.

The integrated intensity of the Coulomb curve between 150-200 a.u. as a function of pump-probe delay is shown in Figure S1 (blue). An error function was fit to the onset of this rise by fitting against the pre-time zero baseline and the asymptotic intensity. The midpoint of this rise then defines our experimental time-zero to which all of our plots in the main paper and supplementary information are referenced to. The results of the calculation are shown in red. Changing the integration range shifts this time zero by approximately 40 fs at most therefore we assign a cautious uncertainty to our value of ±50 fs.

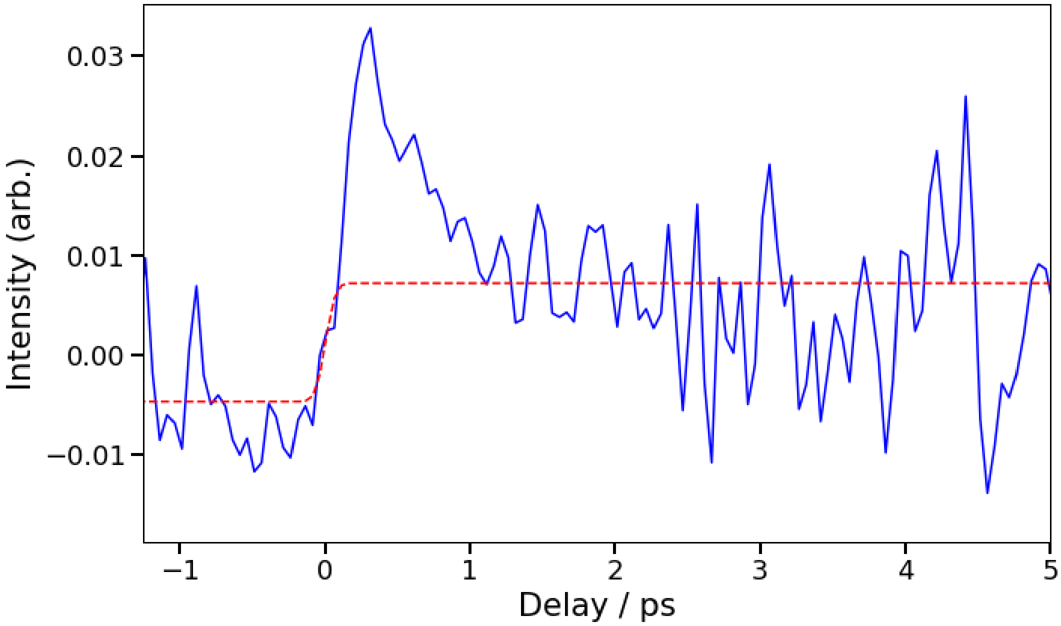

FIG. S1: Integrated intensity between 150-200 a.u. of the residual between experimental data and fit as a function of pump-probe delay (blue). An error function fit shown in red.

II. MASS SPECTRA COVERING FULL M/Z RANGE

Figure S2 presents two sets of two mass spectra obtained for a) 2IT and b) 3IT these are analogous to those presented in Figure 1 of the main manuscript for 2IT but cover the m/z range of the parent ion peak which was not included in the main manuscript paper as it does not form part of the discussion. The spectra obtained from the two isomers are similar in structure. The broad peaks observed in the mass spectra are due to fragments formed with a wide range of momenta following ionisation and Coulomb explosion. The spectrum obtained from the combined effect of the UV and XUV pulses, which is averaged over all positive pump-probe delays, highlights that the main changes induced by the pump-pulse are localised to the iodine fragment ions. The UV pump results in an increased intensity of the I^{n+} peaks over a very narrow m/z range, indicating that these fragments are released with a much lower spread of momenta.

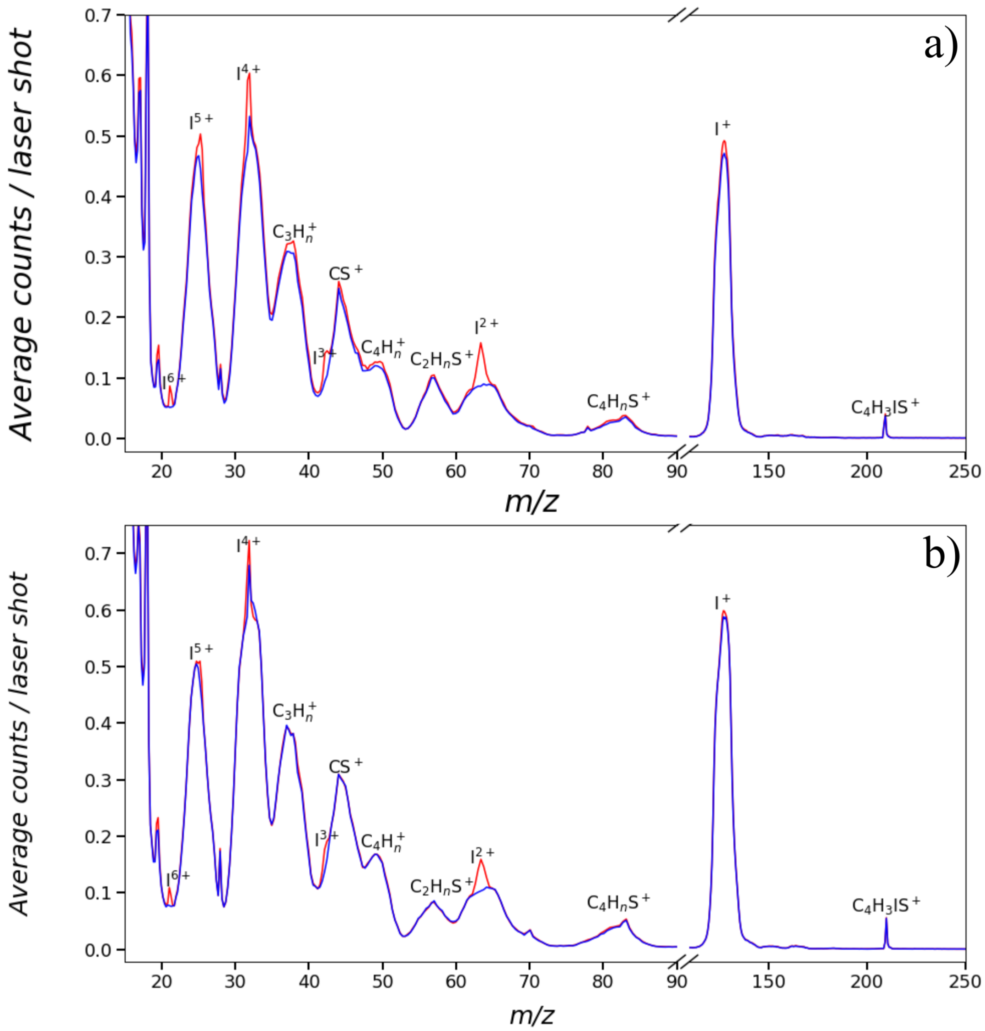

FIG. S2: Mass spectra obtained from ionisation of 3IT by UV + XUV pulses (blue) and by XUV pulses only (red). Each spectrum has been normalised by the total number of shots. The UV + XUV signal has been summed over all positive pump-probe delays (i.e. with the UV pulse preceding the XUV pulse).

III. UV ONLY MASS SPECTRA

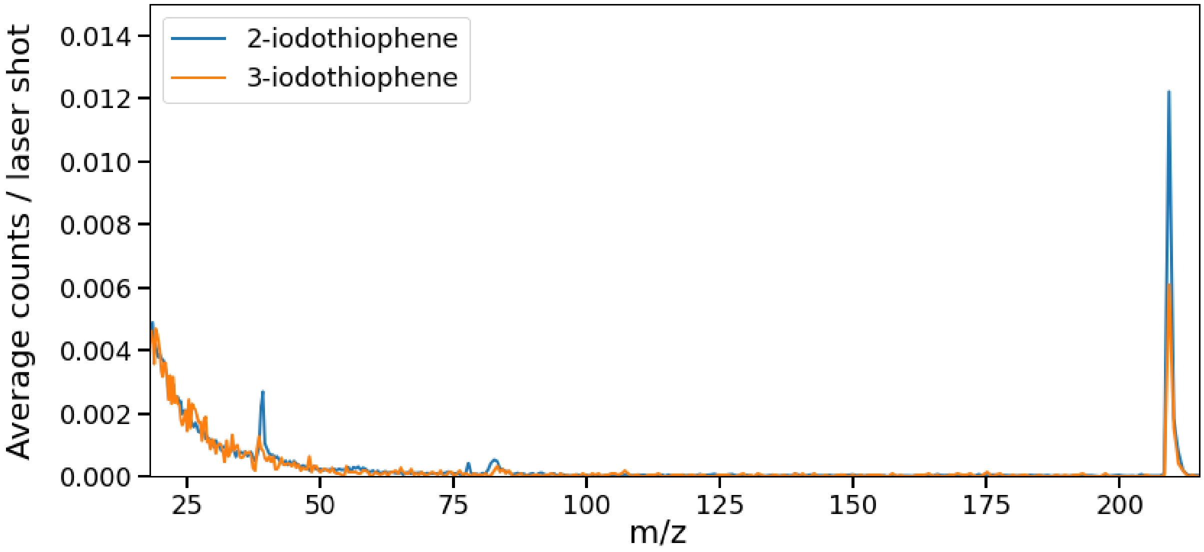

FIG. S3: Mass spectra obtained from ionisation of 2IT (blue) and 3IT (orange) by UV pulses. Each spectrum has been normalized by the total number of shots.

Figure S3 shows the mass spectra obtained following ionization of 2IT and 3IT by only the 262 nm UV pulse. A sharp peak is visible just below 40 mz which can be attributed to ionization of background gas. A small signal associated with the ionized parent molecule is also visible. Overall UV only produces signals averaging less than 0.005 counts per laser shots, compared to as high as 0.7 counts per laser shot visible in Figure S2. This means any contribution from UV only ionization can be treated as negligible with respect to the discussed pump-probe signals.

IV. MOMENTUM MAPS

Figures S3 and S4 summarise the time-resolved difference (XUV only contribution subtracted) momentum distributions as a function of pump-probe delay of the I^{3+} and I^{4+} ions from UV photolysis of 2IT and 3IT, respectively. Resolution decreases as a function of iodine charge state due to increasing overlap with signal from other ions. Iodine charge states +5 and above were not able to be resolved. The features present in the spectra of I^{3+} and I^{4+} are similar to I^{2+} as shown and discussed in the main manuscript. No additional features are found, however the decreasing resolution prevents detection of the lower energy pump-probe feature. The global fit used in the main manuscript is simplified to only two components, the ground state depletion and prompt dissociation.

As discussed in the main manuscript the iodine atom produced by the neutral dissociation process may only be observed once the iodine fragment has reached a critical distance, d_c, beyond which CT can no longer occur. A classical OTB model was used to calculate this critical distance. This states that charge may only be transferred if the Coulombic barrier to electron transfer is lower than the ionisation energy of the site 'donating' the electron. It is important to note that d_c will increase as the charge on the iodine fragment increases. The dissociation velocity does not change therefore a shift towards later detection time is expected for iodine in higher charge states. For an electron transfer from an iodine ion to a neutral ring co-fragment (C_4H_3S), d_c is given by:

$$d_c = \frac{1 + 2\sqrt{n}}{IP_{\mathrm{C_4H_3S}}} \tag{1}$$

where n is the iodine charge state and $IP_{\mathrm{C_4H_3S}}$ is the ionisation energy of the thiophenyl radical. There are no direct measurements of the radical's ionisation energy. We calculate an estimate using Hess' Law and previously reported values for: IP of thiophene (8.87 eV)[1], appearance threshold for forming $C_4H_3S^+$ + H (13.05 ± 0.05 eV) [1, 2], and

C-H bond strength in thiophene (4.99 ± 0.13 eV)[3, 4]. This gives an $IP_{\mathrm{C_4H_3S}}$ value of approximately 8.06 eV. A study on furan (the oxygen analogue of thiophene) found the difference between $\alpha-$ and $\beta-$C-H bond strengths to be negligible[5], therefore this value remains the same for both isomers. The expected appearance time is calculated using the critical distance obtained in this manner and the average asymptotic momentum of observed features. This approach assumes the C-I bond is broken promptly and the asymptotic velocity is reached instantaneously.

The expected appearance values from this model are compared with the observation times obtained from the fit in Table I and show the expected increase in observation time with increasing charge state. The agreement is generally good for both isomers which validates the estimated value of the ionisation energy of the thiophenyl radical as well as the time-zero determination. The results related to 3IT show generally bigger discrepancy than those of 2IT, the deviations fall within expected uncertainty therefore it is unlikely the differences hint at some deviation from the proposed prompt dissociation model.

Iodine charge state	I2+	I3+	I4+
2IT	267	297	338
3IT	264	268	305
OBT predicted	248	290	338

TABLE I: Values of appearance time of higher KE pump-probe feature obtained from the fitting to the experimental data following photolysis of 2IT and 3IT, and the equivalent times predicted by the OBT model.

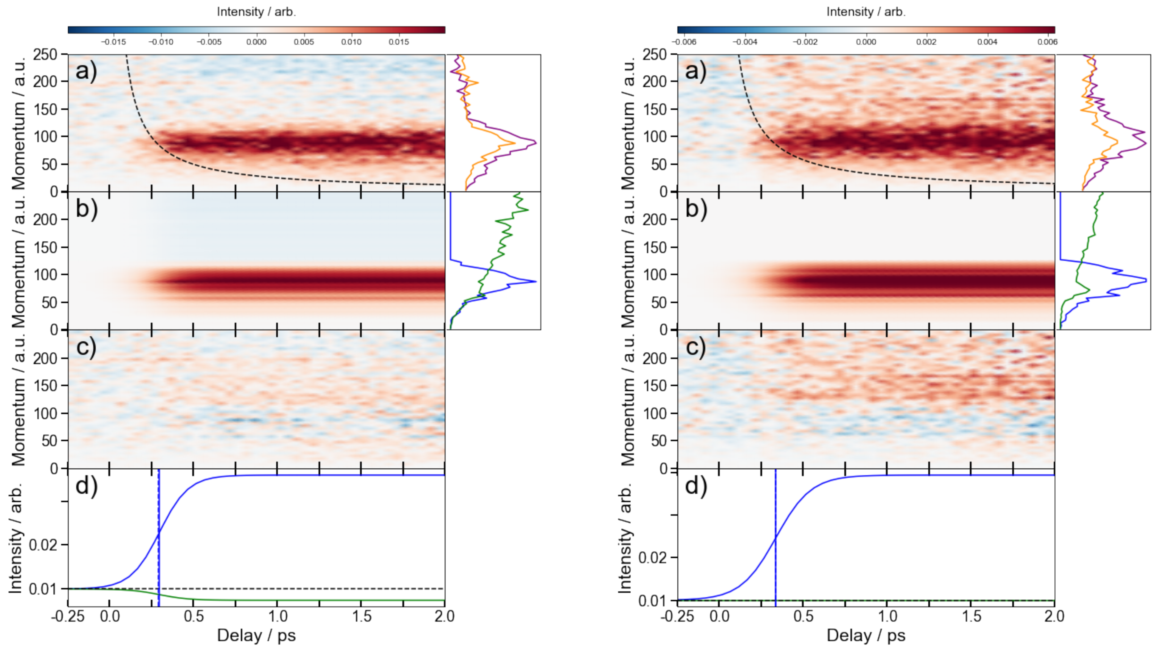

FIG. S4: a) Time-resolved difference map of the momentum of the I^{3+} (left) and I^{4+} (right) fragments formed by 262 nm photolysis of 2IT and subsequent XUV probing (i.e. after subtracting the ground state (XUV only) contribution). The intensity vs momentum distributions at early (200-500 fs) and late (>3 ps) pump-probe delays are shown at the far right (plotted as orange and purple lines, respectively. b) Fit to the experimental data using a time-varying sum of the two intensity vs momentum basis functions shown at the far right, as described in the main manuscript. The two basis functions are displayed with a common peak intensity. c) Residual error between the experimental and fitted data. d) Time-dependent amplitudes of the basis functions obtained from the fit. The appearance time obtained in the fit is shown as a solid vertical blue line and the expected appearance time from OBT model is shown as dashed blue line.

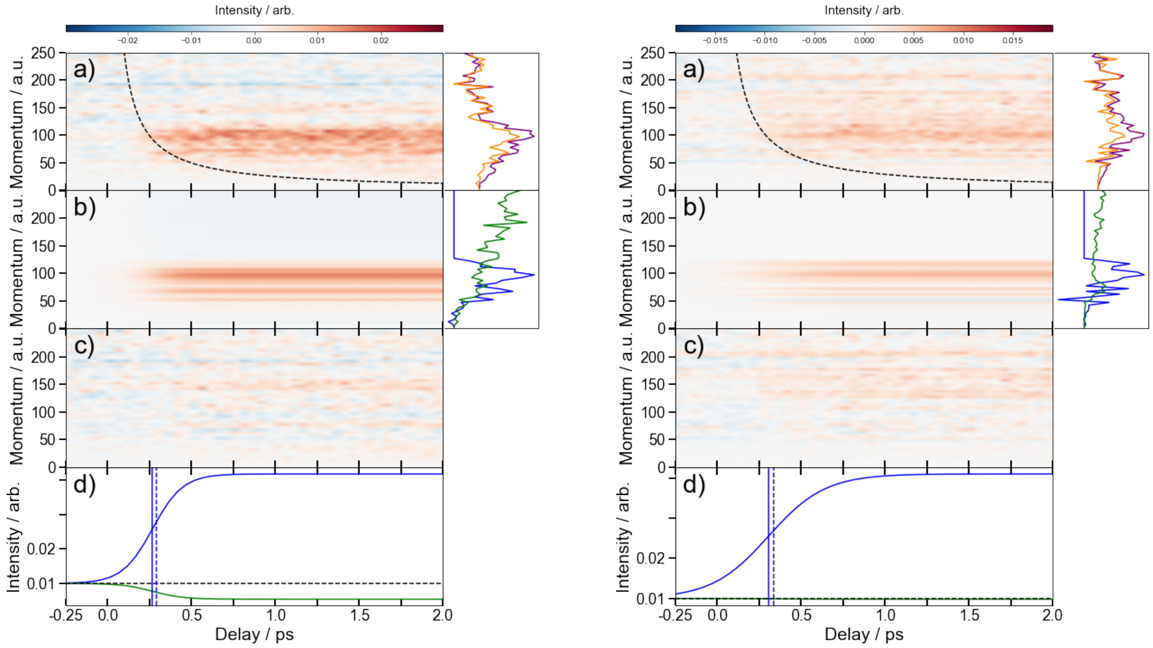

FIG. S5: a) Time-resolved difference map of the momentum of the I^{3+} (left) and I^{4+} (right) fragments formed by 262 nm photolysis of 3IT and subsequent XUV probing (i.e. after subtracting the ground state (XUV only) contribution). The intensity vs momentum distributions at early (200-500 fs) and late (>3 ps) pump-probe delays are shown at the far right (plotted as orange and purple lines, respectively. b) Fit to the experimental data using a time-varying sum of the two intensity vs momentum basis functions shown at the far right, as described in the main manuscript. The two basis functions are displayed with a common peak intensity. c) Residual error between the experimental and fitted data. d) Time-dependent amplitudes of the basis functions obtained from the fit. The appearance time obtained in the fit is shown as a solid vertical blue line and the expected appearance time from OBT model is shown as dashed blue line.

V. GLOBAL FIT BASIS FUNCTIONS

The basis functions used in the fit presented in the main paper are reproduced in Figure S6 to allow easier comparison of the relative widths and positions.

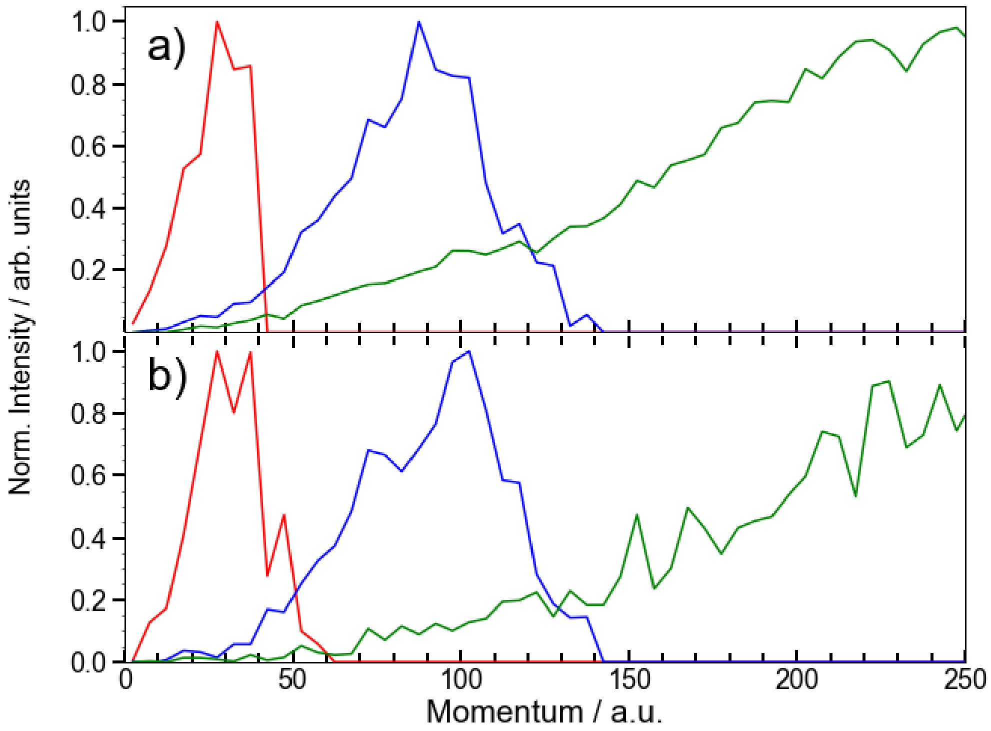

FIG. S6: The basis functions used in the adapted global fit described in the main manuscript. Panels a) and b) show basis functions associated with photolysis of 2IT and 3IT respectively. The basis functions are normalized to their peak intensity. Red curve describes the delayed pump-probe feature, blue describes the prompt feature, and green describes the ground state Coulomb explosion.

VI. EXPERIMENTAL AND FIT TRANSIENTS

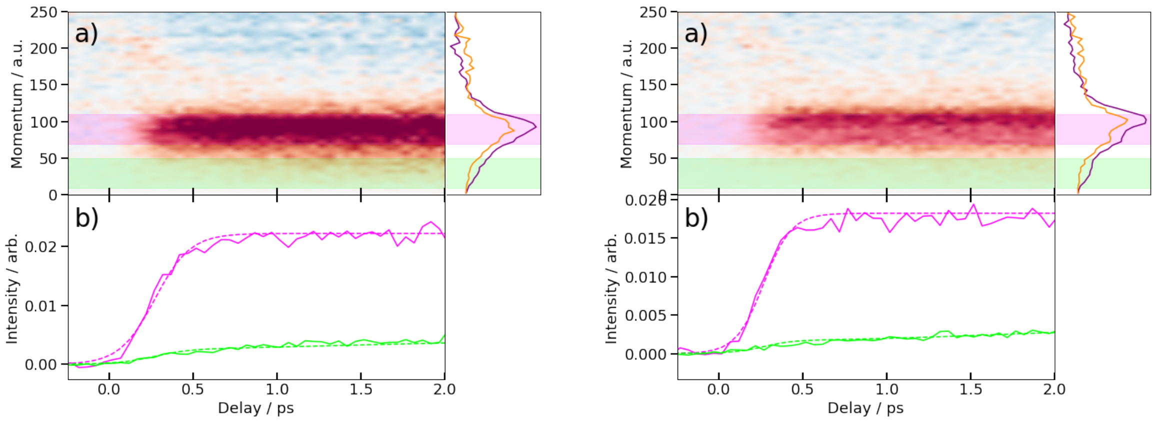

FIG. S7: a) Time-resolved difference map of the momentum of the I^{2+} fragments formed by 262 nm photolysis of 2IT (left) and 3IT (right) followed by XUV probing (i.e. after subtracting the ground state (UV late) contribution). The intensity vs momentum distributions at early (200-500 fs) and late (>3 ps) pump-probe delays are shown at the far right (plotted as orange and purple lines, respectively. Pink and lime-colored boxes are plotted to highlight integration regions presented in next panel. b) Integrated transients in regions: 10-50 a.u. (lime) and 80-130 a.u. (pink) of the experimental difference map (solid lines) as well as the result of the fit as described in the main manuscript (dashed lines).

Figure S7 compares the integrated transients from experimentally acquired data as well as the fitted equivalent for 2IT and 3IT. The two integration regions represent the 'prompt' and 'delayed' channels. A prompt and delayed rise can be observed in panels b) of the figure, and both curves are matched well by the model used as described in the main manuscript. This match provides validity to the model, the results of which are able to provide much more insight to to these two significantlly overlapped functions.

VII. UV ABSORPTION SPECTRUM MEASUREMENTS

The gas-phase UV absorption spectra reported in Fig. 5 of the main manuscript were recorded using a Cary 50 UV-Vis spectrometer. A few drops of liquid sample was added to a quartz cell with 1 cm path length, and the absorption spectra of the vapor were recorded, with approximately 1.5 nm spectral resolution.

---

[1] E. Rennie, L. Cooper, L. Shpinkova, D. Holland, D. Shaw, and P. Mayer, International Journal of Mass Spectrometry **290**, 142 (2010).
[2] J. Butler and T. Baer, Journal of the American Chemical Society **102**, 6764 (1980), https://doi.org/10.1021/ja00542a016.
[3] L. M. Culberson, C. C. Blackstone, A. A. Wallace, and A. Sanov, The Journal of Physical Chemistry A **119**, 9770 (2015), https://doi.org/10.1021/acs.jpca.5b04672.

[4] L. M. Culberson and A. Sanov, The Journal of Chemical Physics **134**, 204306 (2011), https://pubs.aip.org/aip/jcp/article-pdf/doi/10.1063/1.3593275/13538846/204306_1_online.pdf.
[5] J. A. DeVine, M. L. Weichman, S. J. Lyle, and D. M. Neumark, Journal of Molecular Spectroscopy **332**, 16 (2017).
